# Lowered oxidative capacity in spinal muscular atrophy, Jokela type; comparison with mitochondrial muscle disease

**DOI:** 10.3389/fneur.2023.1277944

**Published:** 2023-11-08

**Authors:** Nadja Ratia, Edouard Palu, Hanna Lantto, Emil Ylikallio, Ritva Luukkonen, Anu Suomalainen, Mari Auranen, Päivi Piirilä

**Affiliations:** ^1^Unit of Clinical Physiology, HUS Medical Diagnosis Center, University of Helsinki and Helsinki University Hospital, Helsinki, Finland; ^2^Unit of Neurophysiology, HUS Medical Diagnosis Center, University of Helsinki and Helsinki University Hospital, Helsinki, Finland; ^3^Clinical Neurosciences, Neurology, University of Helsinki and Helsinki University Hospital, Helsinki, Finland; ^4^Stem Cells and Metabolism Research Program, Faculty of Medicine, University of Helsinki, Helsinki, Finland; ^5^Finnish Institute of Occupational Health, Helsinki, Finland; ^6^Research Program of Stem Cells and Metabolism, Faculty of Medicine, University of Helsinki, Helsinki, Finland; ^7^Neuroscience Center, HiLife, University of Helsinki, Helsinki, Finland

**Keywords:** spinal muscular atrophy Jokela type (SMAJ), motoneuron disease, mitochondrial myopathy, cardiopulmonary exercise testing, lactate

## Abstract

**Introduction:**

Spinal muscular atrophy, Jokela type (SMAJ) is a rare autosomal dominantly hereditary form of spinal muscular atrophy caused by a point mutation c.197G>T in *CHCHD10*. *CHCHD10* is known to be involved in the regulation of mitochondrial function even though patients with SMAJ do not present with multiorgan symptoms of mitochondrial disease. We aimed to characterize the cardiopulmonary oxidative capacity of subjects with SMAJ compared to healthy controls and patients with mitochondrial myopathy.

**Methods:**

Eleven patients with genetically verified SMAJ, 26 subjects with mitochondrial myopathy (MM), and 28 healthy volunteers underwent a cardiopulmonary exercise test with lactate and ammonia sampling. The effect of the diagnosis group on the test results was analysed using a linear model.

**Results:**

Adjusted for sex, age, and BMI, the SMAJ group had lower power output (*p* < 0.001), maximal oxygen consumption (VO_2_ max) (*p* < 0.001), and mechanical efficiency (*p* < 0.001) compared to the healthy controls but like that in MM. In the SMAJ group and healthy controls, plasma lactate was lower than in MM measured at rest, light exercise, and 30 min after exercise (*p* ≤ 0.001–0.030) and otherwise lactate in SMAJ was lower than controls and MM, in longitudinal analysis *p* = 0.018. In MM, the ventilatory equivalent for oxygen was higher (*p* = 0.040), and the fraction of end-tidal CO_2_ lower in maximal exercise compared to healthy controls (*p* = 0.023) and subjects with SMAJ.

**Conclusion:**

In cardiopulmonary exercise test, subjects with SMAJ showed a similar decrease in power output and oxidative capacity as subjects with mitochondrial myopathy but did not exhibit findings typical of mitochondrial disease.

## 1. Introduction

Spinal muscular atrophy, Jokela type (SMAJ), also known as late-onset spinal motor neuronopathy (LOSMoN), is autosomal dominantly inherited spinal muscular atrophy caused by Finnish founder mutation c.197G>T (Gly66Val) in *CHCHD10* gene. Common symptoms of SMAJ include muscle weakness, fasciculation, and cramps concentrating in the lower limbs (1–3).

CHCHD10 belongs to a family of mitochondrial proteins that have been shown to be involved in the regulation of mitochondrial function and oxidative phosphorylation (4, 5). Mutations in *CHCHD10 gene* are also associated with neurodegenerative disorders, such as amyotrophic lateral sclerosis (ALS), axonal Charcot Marie Tooth disease, and frontotemporal dementia, as well as some forms of autosomal dominantly hereditary mitochondrial disease (6–9). SMAJ manifests as a motoneuron disease with skeletal muscle damage indicated, e.g., by the rise in creatine kinase hallmarking the disease (1, 10). In SMAJ, although muscle biopsy findings suggest that mitochondrial pathology in SMAJ would been minimal, in metabolic examinations suggestions on mitochondrial dysfunction have been found based on changes of pyruvate and succinate levels (10) as well as in magnetic resonance measurements based on decreased mitochondrial ATP synthesis function during exercise (11).

On the other hand, primary mitochondrial myopathies are a diverse group of disorders associated with changes in mitochondrial structure and function, leading to dysfunction of oxidative phosphorylation, thus impairing cellular energy metabolism, and typically manifest with muscle symptoms like fatigue, muscle weakness, and exercise intolerance. mitochondrial myopathy, encephalopathy, lactic acidosis, and stroke-like episode syndrome (MELAS) is the most common maternally inherited mitochondrial disease, and caused by the m.3243>G mutation in the MT-TL1-gene encoding the mitochondrial tRNA [Leuk (UUR)]. The m.3243A>G mtDNA mutation causes a variability of phenotypes with variable degrees of myopathy. Another mitochondrial myopathy, chronic progressive external ophthalmoplegia (CPEO), is more genetically heterogenous, with around 50% of the cases caused by sporadic deletion of the mtDNA. Usually, only a portion of the patient’s mitochondria carry the mutated mtDNA, and the concentrations of the defective mitochondria differ between different tissues and varying severity of symptoms in patients carrying the same mtDNA mutation with the variability not explained by mutation amount (12–15).

Elevated lactate concentrations, both at rest and during exercise, are typical for mitochondrial myopathies, and excessive ventilation during exercise has been reported, and cardiopulmonary exercise testing with lactate samples has been used as a part of their diagnostic palette (16–19). As a non-invasive tool, cardiopulmonary exercise testing also provides information about the different causes of exercise intolerance as well as the subject’s cardiac and pulmonary capacity, which can be especially useful as exercise intolerance and muscle fatigue are common symptoms in the population at large.

We aimed to characterize the oxidative capacity of subjects with SMAJ compared to healthy controls. In addition, we compared the results of patients with SMAJ with subjects with genetically verified mitochondrial myopathy to see if the stress associated with exercise testing would cause physiological findings of mitochondrial myopathy also in SMAJ.

## 2. Methods

### 2.1. Subjects

The study subjects were 11 patients with genetically verified SMAJ (4 men and 7 women) who had previously taken part in a biomarker study (10), 26 subjects with mitochondrial disease genetically verified from either blood or muscle samples (9 men and 17 women), and 28 healthy volunteers (11 men and 17 women). Of the subjects with mitochondrial disease, 15 subjects were diagnosed with CPEO (8 with multiple mitochondrial DNA deletions and 7 with a single deletion) and 11 with MELAS (10 of them with mutation m.3243A>G and 1 with m.3302A>G). All patients with SMAJ and all mitochondrial disease patients except one with CPEO had exhibited muscle symptoms, but not severe enough to prevent an exercise test. All patients with SMAJ and healthy subjects underwent a cardiopulmonary exercise test complemented with lactate, ammonium, and venous blood gas samples. The subjects with mitochondrial disease had previously undergone a similar test as part of their diagnostic work-up.

All the subjects gave their written informed consent to participate in the study. The study was undertaken according to the Declaration of Helsinki. Helsinki University Hospital’s medical ethics review board has approved the study: 314/13/03/01/2008, 199/13/3/01/2011, and HUS/2084/2020.

### 2.2. Exercise test

Work-conducted maximal cardiopulmonary exercise test with gas exchange analysis (spiroergometry) was performed using methods described previously (20, 21). For measurement of respiratory gases, a tightly secured facemask (Rudolph series 7910, Hans Rudolph Inc., United States) was used, with a dead space varying between masks, small, medium, or large, according to the manufacturer’s information. After about 10 min of rest, the subject sat on the bicycle, and the breath gas recording was started. One minute of rest breathing was recorded before the exercise while the test subject was sitting upright, breathing through a breath-by-breath gas exchange analysis system (Vyntus CPX in 2017-202m SensorMedics, Yorba Linda CA, United States). Breath gas recording continued throughout the exercise test, and 30 s mean values of breath gases were reported. The exercise test was performed using an electrically braked bicycle ergometer (Ergoselect 200P, Ergoline Gmbh, Bitz, Germany). The starting workload was 40 W for women and 50 W for men, and the load was increased at 3 min intervals by 40 or 50 W, respectively. The exercise was continued until hard exertion (17–20/20 scale of perceived exertion), and the respiratory exchange rate (RER = V′CO_2_/V′O_2_) of at least 1.0 was reached. The ventilatory threshold was assessed at the point of slope change of V′CO_2_ to increase more steeply with respect to V′O_2_ (V-slope method) (22). Mechanical efficiency was calculated when maximal power (W) was converted into calories, and this was divided by the maximal oxygen consumption, also converted into calories.

Blood pressure was measured manually from the right arm using a stethoscope and a sphygmomanometer (Erka) before, during, and 3 and 6 min after exercise. A 12-lead ECG was continuously monitored during the exercise test using a digital ECG device and recorded using a computerized device (CardioSoft version V6-5, and later version 7-7.0851, GE Medical Systems, Milwaukee, WI, United States). Mason-Likar leads were used during the exercise. Peripheral arterial oxygen saturation (SpO_2_) was measured with two pulse oximeters (MySignS, EviteC, NJ, United States). The sensors were attached to the subject’s earlobe giving a reliable signal and their left middle finger.

A cannula was inserted in a vein in the subject’s left antecubital fossa. Through this, samples of venous blood were collected for analysis of blood gases, lactate, and ammonia (NH_4_^+^) before exercise (rest), during the first exercise step (light exercise), and during maximal exercise as well as 2, 4, 6, 10, 20, and 30 min after exercise.

The blood gas samples were analysed using a Radiometer ABL90 or ABL825 analyser (Radiometer Medical, Copenhagen, Denmark) in the central laboratory of the hospital. For the SMAJ and healthy control of groups, the lactate and ammonia samples were analysed with Siemens Atellica^®^ Solution Chemistry analyser (Siemens Healthineers, Erlangen, Germany). For the mitochondrial disease group, three different automated routine clinical chemistry platforms were in clinical use to analyse lactate and ammonia samples during the time when results were collected [Cobas Integra^®^ 400 chemistry analyser (Roche Modular, Roche Diagnostics, Basel, Switzerland), Abbott^™^ Architect^™^ c16000 or c8000 (Abbott Laboratories, Abbott Park, IL, United States), Siemens Atellica^®^ Solution Chemistry analyzer (Siemens Healthineers, Erlangen, Germany)]. Before changing the instruments and assays the lactate and ammonia assays were verified for clinical laboratory use using internal quality control materials as per normal quality practice. As the ammonia values observed during the cardiopulmonary exercise test are considerably lower than the standard quality controls, for the study’s purposes the reproducibility of the assay was also estimated using internal quality control material, and trueness in relation to the previous assay was estimated using patient samples from the healthy controls in an internal assessment as reported earlier (23). In this internal assessment there were no changes in the lactate results between these analysis methods, but a significant change in ammonia values. Due to the difference in the analysis method, the ammonia results for the MELAS/CPEO group were excluded from the analysis.

### 2.3. Electrodiagnostic testing

All the SMAJ patients had electrodiagnostic testing (ENMG) consisting of nerve conductions studies (NCS) and needle electromyography (EMG) performed. We performed ENMG on 5 MELAS/PEO patients, and for 4, we reviewed the ENMG reports of studies performed earlier by other neurophysiologists. For one MELAS/PEO patient, the report could not be obtained as the study had been performed a very long time ago. The severity of the motor neuropathy was graded according to a three-level scale as either mild, moderate, or severe. The classification was primarily based on the neurophysiologist’s assessment of motor unit loss in limb muscles, but the presence or absence of fibrillation potentials, indicative of ongoing denervation, was also assessed, with fibrillation potentials present in at least some distal muscles in moderate and severe motor neuropathy but absent in mild motor neuropathy.

The findings of sensory neuropathy were similarly graded into normal, mild, moderate or severe, though severe neuropathy (absent response in upper extremities) was not observed. Mild sensory neuropathy was defined as diminished sensory amplitudes in the lower limbs, while in moderate sensory neuropathy sensory responses were absent in lower limbs.

### 2.4. Statistical analysis

The data was analyzed using IBM SPSS 27 Statistics program. A linear model was utilized to assess the diagnosis groups’ effects on the cardiopulmonary exercise test results, plasma lactate, and ammonia concentrations, and blood gas results, adjusted for age, sex, and BMI.

Repeated samples two-way ANOVA was also calculated for the lactate and ammonia samples from the resting state on.

The statistical significance threshold for the *p*-value was <0.05.

## 3. Results

### 3.1. Cardiopulmonary exercise testing results and patient characteristics

The age, sex, genetics, symptoms, laboratory, and neurophysiological findings of the subjects with SMAJ are presented in Table 1. Anthropometric data and the results of the cardiopulmonary exercise testing per diagnosis group and sex are collected in Table 2, showing higher results in men than in women in most measured values, as could be expected. The SMAJ group was slightly older than the healthy controls and the MELAS/CPEO group. Due to the rarity of the diseases and to better match the sex and age distribution of the groups, and as the exercise parameters are heavily age and sex-dependent, we included both CPEO and MELAS patients in the mitochondrial disease control group. The age range for the SMAJ group was 32–76 years (mean 55.97 SD 14.7), for MELAS/CPEO 16–71 years (mean 44.4 SD 14.7), and for the controls 22–76 years (mean 50.0 SD 18.2).

**Table 1:** The age, sex genetic basis, symptoms, laboratory and neurophysiological findings of the subjects with SMAJ.

Patient	Sex	Age at test	Genetic variant	Symptoms	Age at onset of symptoms	Duration of symptoms	Muscle biopsy results (if available)	ENMG	Creatine kinase	Resting lactate	Lower-limb muscle MRI results
1	F	41	*CHCHD10* c. 197G>T p.(Gly66Val)	-Fasciculation, cramps -Upper limb symptoms +	35	6	—	-Mild motor neuropathy -Sensory neuropathy + (mild)	122–283	0.7	Atrophy −
2	F	61	*CHCHD10* c. 197G>T, p.(Gly66Val)	-Lower limb weakness -Clumsiness -Upper limb symptoms +	50	11	—	-Moderate motor neuropathy -Sensory neuropathy −	286–299	1.0	—
3	F	49	*CHCHD10* c. 197G>T p.(Gly66Val)	-Lower limb weakness -Cramps, fasciculation, pain -Upper limb symptoms −	40	9	—	-Severe motor neuropathy -Sensory neuropathy −	374–483	0.7	Atrophy +
4	F	72	*CHCHD10* c. 197G>T p.(Gly66Val)	-Lower limb muscle pain -Upper limb symptoms −	51	21	-Denervation atrophy -Myopathy -A few COX-negative strands	-Moderate motor neuropathy -Sensory neuropathy −	205–604	0.8–1.0	Atrophy+
5	F	76	*CHCHD10* c. 197G>T p.(Gly66Val)	-Lower limb weakness -Numbness -Upper limb symptoms −	50	26	—	-Severe motor neuropathy -Sensory neuropathy + (mild)	136	1.5–2.7	—
6	F	66	*CHCHD10* c. 197G>T p.(Gly66Val)	-Lower limb weakness -Cramps -Upper limb symptoms +	40	26	-Severe denervation atrophy -Secondary myopathy -A few COX-negative strands	-Severe motor neuropathy -Sensory neuropathy −	153–204	0.9	Atrophy +
7	M	71	*CHCHD10* c. 197G>T p.(Gly66Val)	-Lower limb weakness -Clumsiness -Upper limb symptoms +	55	16	—	-Moderate motor neuropathy -Sensory neuropathy + (moderate)	239–389	0.9	—
8	F	54	*CHCHD10* c. 197G>T p.(Gly66Val)	-Lower limb weakness -Cramps -Upper limb symptoms +	47	7	—	-Severe motor neuropathy -Sensory neuropathy−	269–299	0.6	—
9	M	32	*CHCHD10* c. 197G>T p.(Gly66Val)	-Lower limb muscle pain, cramps -Upper limb symptoms −	30	2	—	-Moderate motor neuropathy -Sensory neuropathy −	320	1.2	—
10	M	38	*CHCHD10* c. 197G>T p.(Gly66Val)	-Lower limb weakness -Cramps, fasciculation -Upper limb symptoms +	32	6	—	-Mild motor neuropathy -Sensory neuropathy + (mild)	131	0.9	
11	M	54	*CHCHD10* c. 197G>T p.(Gly66Val)	-Cramps, fasciculation -Numbness -Upper limb symptoms +	51	3	—	-Mild motor neuropathy -Sensory neuropathy −	351	1.0	—

**Table 2:** Cardiopulmonary exercise test results per diagnosis group and sex.

	Men			Women		
	SMAJ mean (SD) *n* = 4	MELAS/CPEO mean (SD) *n* = 9	Controls mean (SD) *n* = 11	SMAJ mean (SD) *n* = 7	MELAS/CPEO mean (SD) *n* = 17	Controls mean (SD) *n* = 17
Age (years)	48.5 (17.1)	45.0 (12.7)	45.8 (18.2)	59.9 (12.6)	44.0 (16.1)	52.8 (18.2)
Height (cm)	174.6 (3.3)	172.7 (6.3)	183.5 (6.5)	163.2 (4.6)	164.4 (4.4)	161.6 (6.2)
Weight (kg)	77.1 (8.8)	74.1 (12.3)	73.4 (11.5)	64.3 (10.3)	61.8 (11.3)	64.3 (9.9)
BMI	25.3 (2.7)	24.7 (2.6)	24.0 (3.0)	24.2 (4.1)	22.9 (4.1)	24.5 (3.0)
**Rest**						
Systolic pressure, rest (mmHg)	118.5 (7.6)	140.9 (26.5)	129.5 (14.2)	140.9 (20.4)	130.1 (24.3)	130.5 (21.4)
Diastolic pressure, rest (mmHg)	72.5 (7.0)	84.0 (17.3)	74.9 (8.0)	84.0 (7.1)	81.5 (12.6)	76.4 (11.1)
Heart rate (1/min)	63.6 (9.1)	75.9 (12.4)	65.0 (8.0)	69.6 (12.1)	75.8 (16.9)	70.7 (9.3)
V′O_2_ (L/min)	0.41 (0.12)	0.34 (0.09)	0.43 (0.02)	0.29 (0.09)	0.33 (0.13)	0.30 (0.06)
V′O_2_/kg (mL/min/kg)	5.4 (1.6)	4.1 (1.1)	5.3 (1.6)	4.8 (1.5)	5.4 (2.1)	4.7 (1.0)
RER, rest	0.74 (0.05)	0.75 (0.09)	0.78 (0.08)	0.77 (0.04)	0.81 (0.08)	0.80 (0.07)
**Maximal exercise**						
Syst. pressure (mmHg)	216.0 (28.9)	207.4 (29.0)	207.2 (9.4)	187.3 (23.6)	177.4 (18.8)	190.9 (24.4)
Diast. Pressure (mmHg)	92.0 (18.8)	99.4 (14.9)	82.0 (17.6)	99.3 (12.0)	86.1 (28.1)	89.7 (14.4)
Heart rate (bpm)	163.5 (25.3)	166.7 (17.0)	166.7 (16.9)	148.6 (32.4)	158.1 (18.2)	166.0 (14.8)
Heart rate (bpm)/perd. maximum	90.0 (11.3)	91.4 (9.0)	91.4 (7.8)	84.2 (15.9)	91.8 (24.2)	93.8 (5.6)
RER, max	1.18 (0.06)	1.22 (0.11)	1.16 (0.07)	1.14 (0.10)	1.21 (0.12)	1.18 (0.09)
Breathing frequency, (1/min)	33.0 (4.1)	35.0 (7.1)	37.3 (7.8)	33.6 (8.1)	37.8 (8.9)	36.2 (5.8)
Ventilatory threshold (L/min)	1.12 (0.14)	1.20 (0.38)	1.70 (0.53)	0.91 (0.23)	1.04 (0.36)	0.91 (0.23)
W_max_/3 (W)	162.5 (43.7)	156.0 (37.1)	232.4 (51.6)	75.1 (47.7)	101.7 (41.3)	131.8 (39.8)
W_max_/3 min (% of predicted value)	84.68 (11.1)	81.7 (20.7)	117.4 (13.7)	66.0 (37.8)	73.5 (25.4)	100.4 (18.6)
W_max_/VO_2_%	20.5 (3.0)	20.2 (2.3)	22.4 (1.4)	15.4 (4.6)	18.6 (3.5)	20.6 (2.3)
V′O_2_ max (L/min)	2.25 (0.34)	2.27 (0.60)	2.98 (0.64)	1.31 (0.46)	1.54 (0.51)	1.78 (0.45)
V′O_2_/kg max (mL/min/kg)	29.1 (5.9)	30.2 (4.9)	37.4 (9.7)	20.9 (8.2)	25.6 (9.8)	27.2 (7.3)
V′O_2_/HR	13.9 (0.9)	13.5 (3.2)	17.9 (3.1)	9.0 (1.5)	9.8 (3.0)	10.5 (2.3)
Breathing reserve (%)	30.5 (6.2)	30.0 (11.3)	32.0 (11.5)	34.9 (20.7)	37.7 (13.5)	32.1 (13.0)
V′E/V′O_2_	40.5 (5.3)	40.7 (6.2)	38.1 (5.7)	40.3 (8.1)	43.3 (7.0)	39.2 (6.6)
V′E/V′CO_2_	34.50 (6.1)	33.2 (2.8)	33.0 (4.1)	35.3 (4.7)	36.6 (4.7)	33.8 (4.0)
FETCO_2_ (%)	4.70 (0.57)	4.84 (0.49)	4.90 (0.61)	4.70 (0.78)	4.39 (0.49)	4.90 (0.62)
Tidal volume (L)	2.90 (0.27)	2.66 (0.57)	3.40 (0.58)	1.74 (0.51)	1.91 (0.54)	2.03 (0.33)

SD, standard deviation; SMAJ, spinal muscular atrophy, Jokela type; MELAS/CPEO, mitochondrial disease group; FETCO_2_, fraction of end tidal CO_2_; RER, respiratory exchange rate (V′CO_2_/V′O_2_); V′E, minute ventilation; V′E/V′O_2_, ventilatory equivalent to O_2_; V′E/V′CO_2_, ventilatory equivalent to CO_2_; V′O_2_ max, maximal oxygen uptake; V′O_2_/HR, oxygen pulse; W_max_ 3, maximal power during last three maximal minutes of exercise.

In electromyography (ENMG) used to characterize the subjects, those with SMAJ showed mild to severe motor neuropathy, and four of the patients also presented mild or moderate sensory neuropathy (Table 1). None of the subjects had severe sensory neuropathy, as defined by absent sensory responses in the upper limbs. Of the mitochondrial myopathy patients, ENMG results taken for clinical use were available for 7 MELAS patients and 3 CPEO patients, with 3 of the MELAS patients and all CPEO patients exhibiting myopathic findings. For 4 of the MELAS patients, the results were normal. None of the CPEO or MELAS patients had motor neuropathic or sensory neuropathic findings.

**Figure 1:**
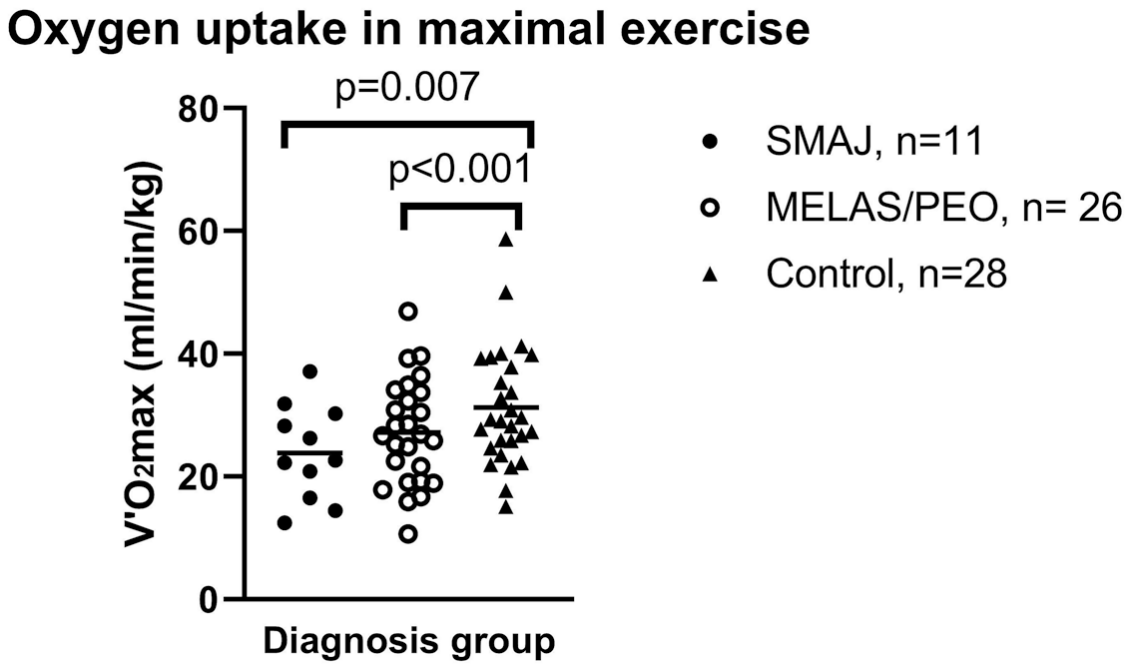
The maximal oxygen uptake during in maximal exercise per weight between diagnosis groups. Mean is displayed as a diagonal line. SMAJ, spinal muscular atrophy, Jokela type; MELAS/PEO, mitochondrial disease group. Statistical significance is presented for linear model results adjusted for age, sex, and BMI.

Adjusted for sex, age, and BMI, the SMAJ group had lower mean power output during the last 3 min of exercise (W_last_ 3) (*p* ≤ 0.001), maximal oxygen consumption (VO_2_ max) (*p* ≤ 0.001), maximal oxygen consumption per kilogram (*p* = 0.007) (Figure 1), oxygen pulse (*p* ≤ 0.001), ventilatory threshold (*p* = 0.045), and mechanical efficiency (W_max_/VO_2_ max) (*p* ≤ 0.001) compared to the healthy controls. Similar differences were also observed between the MELAS/CPEO group and the healthy controls (Table 3). There were no significant differences between SMAJ-group and MELAS/CPEO group in these variables (data not shown).

**Table 3:** Linear model results for the exercise test results per diagnosis group in comparison with the healthy control group adjusted for age, sex, and BMI.

		*B* (SE)	*p*-value
Maximal heart rate	Control	—	Reference
	SMAJ	−8.6 (5.7)	0.129
	MELAS/CPEO	−8.6 (4.4)	0.050
Ventilatory threshold	Control	—	Reference
	**SMAJ**	**−0.24 (0.12)**	**0.040**
	**MELAS/CPEO**	**−0.32 (0.09)**	**<0.001**
RER	Control	—	Reference
	SMAJ	−0.03 (0.03)	0.457
	MELAS/PEO	0.04 (0.03)	0.106
W_max_ 3	Control	—	Reference
	**SMAJ**	**−51.7 (10.9)**	**<0.001**
	**MELAS/CPEO**	**−57.7 (8.4)**	**<0.001**
VO_2_ max	Control	—	Reference
	**SMAJ**	**−0.46 (0.13)**	**<0.001**
	**MELAS/CPEO**	**−0.52 (0.10)**	**<0.001**
VO_2_ max/kg	Control	—	Reference
	**SMAJ**	**−5.3 (1.9)**	**0.006**
	**MELAS/CPEO**	**−6.0 (1.5)**	**<0.001**
VO/HR	Control	—	Reference
	**SMAJ**	**−2.0 (0.9)**	**<0.001**
	**MELAS/CPEO**	**−2.4 (0.6)**	**<0.001**
W_max_/VO_2_%	Control	—	Reference
	**SMAJ**	**−3.60 (0.87)**	**<0.001**
	**MELAS/CPEO**	**−2.65 (0.67)**	**<0.001**
EQO_2_	Control	—	Reference
	SMAJ	0.72 (2.19)	0.741
	**MELAS/CPEO**	**3.46 (1.69)**	**0.041**
EQCO_2_	Control	—	Reference
	SMAJ	1.22 (1.44)	0.398
	MELAS/CPEO	1.95 (1.11)	0.079
FETCO_2_	Control	—	Reference
	SMAJ	−0.18 (0.20)	0.378
	**MELASC/PEO**	**−0.35 (0.16)**	**0.024**
Breathing frequency	Control	—	Reference
	SMAJ	−3.04 (2.54)	0.230
	MELAS/CPEO	−0.16 (1.96)	0.936

*B*, estimated regression coefficient for one-unit increase in continuous variables. SE, standard error; SMAJ, spinal muscular atrophy, Jokela type; MELAS/CPEO, mitochondrial disease group. Statistically significant results are (*p* < 0.05) in bold. FETCO_2_, fraction of end tidal CO_2_; RER, respiratory exchange rate (V′CO_2_/V′O_2_); V′E, minute ventilation; V′E/V′O_2_, ventilatory equivalent to O_2_; V′E/V′CO_2_, ventilatory equivalent to CO_2_; V′O_2_ max, maximal oxygen uptake; V′O_2_/HR, oxygen pulse; W_max_ 3, maximal power during last three maximal minutes of exercise.

The fraction of end-tidal CO_2_ (FETCO_2_) was significantly lower (*p* = 0.023), and the ventilatory equivalent for oxygen (VE/V′O_2_) (*p* = 0.040) higher in the MELAS/CPEO group compared to the healthy controls, which was not observed between the SMAJ group and the healthy controls (Table 3).

For assessing the maximality of exercise, all subjects had respiratory exchange ratio (RER) over 1.0. There was no statistically significant difference between any diagnosis groups in RER or maximal heart rate when adjusted for sex, age, and BMI (Table 3).

### 3.2. Lactate and ammonia

**Figure 2:**
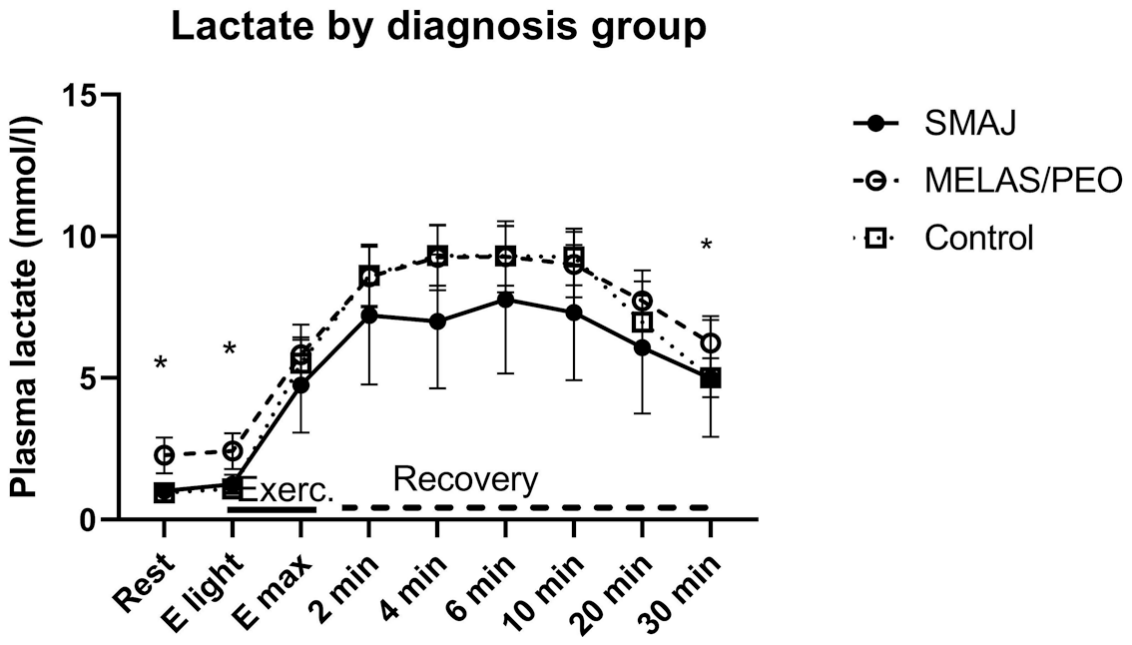
The lactate levels at rest, during exercise, and at recovery phase associated with cardiopulmonary exercise testing. Mean and 95% confidence interval for plasma lactate in the diagnosis groups are given. SMAJ, spinal muscular atrophy, Jokela type; MELAS/PEO, mitochondrial disease group; E light, first exercise step; E_max_, maximal exercise; Exerc., exercise. Statistical significance is presented for linear model results adjusted for age, sex, and BMI with an asterisk (*). Repeated analysis: time*group, *p* = 0.018.

Plasma lactate and ammonia results per diagnosis group are presented in Supplementary Table A1. Adjusted for sex, age, and BMI, we observed a difference in plasma lactate concentration between the healthy controls and MELAS/CPEO group at rest and light exercise (*p* ≤ 0.001) as well as 30 min into the recovery (*p* = 0.020). Similarly, there was a statistically significant difference between the SMAJ and MELAS/CPEO groups at rest and light exercise (*p* ≤ 0.001). Between 2 and 10 min into recovery the lactate values of MELAS/PEO and controls were almost similar and at a higher level than in SMAJ. In repeated samples ANOVA, there was a significant difference in lactate values in relation to time (Wilks’ Lambda *p* < 0.001) and between diagnosis groups in relation to time (Wilks’ Lambda *p* = 0.018) (Supplementary Tables A1, A2) (Figure 2).

**Figure 3:**
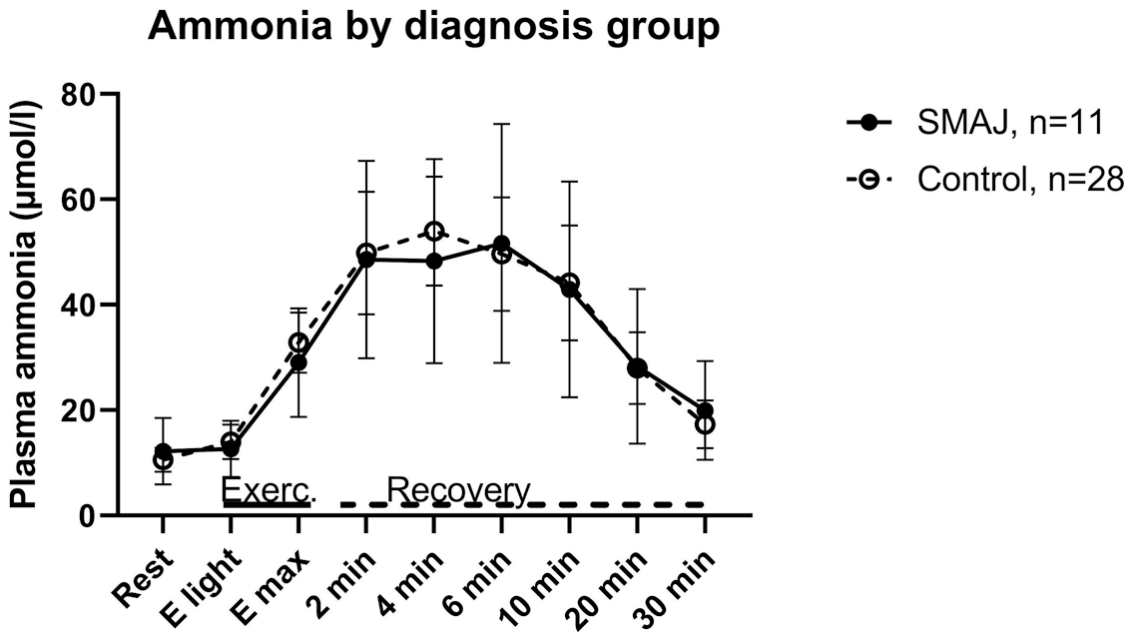
The ammonia levels of the patients with SMAJ and healthy controls at rest, during exercise, and at recovery phase associated with cardiopulmonary exercise testing. SMAJ, spinal muscular atrophy, Jokela type; E light, first exercise step; E_max_, maximal exercise; Exerc., exercise.

As concerns ammonia, there was no statistically significant difference between the SMAJ group and the healthy volunteers in the linear model or between the diagnosis groups in relation to time in the repeated samples ANOVA (Figure 3).

### 3.3. Blood gases

Adjusted for sex, age, and BMI, pH differed 6 min into recovery between the SMAJ group and the healthy controls (*p* = 0.026) and similarly between MELAS/CPEO and the healthy controls (*p* = 0.022). In base excess (BE), there was a significant difference between MELAS/CPEO group and the healthy controls at rest (*p* = 0.028) and light exercise (*p* < 0.001), but no statistically significant changes between the SMAJ group and the healthy volunteers (data not shown).

## 4. Discussion

The present study focuses on analyzing the oxidative capacity and ventilatory profiles of spinal muscular atrophy, Jokela type (SMAJ), which is an autosomal dominant spinal muscular atrophy caused by the Finnish founder mutation c.197G>T (p.Gly66Val) in *CHCHD10*. CHCHD10 is a protein localized in the mitochondrial intermembrane space and has drawn significant attention in recent years due to its association with amyotrophic lateral sclerosis and other neurodegenerative diseases (24). Additionally, variants in the related protein CHCHD2 have been linked to Parkinson’s disease (25).

While *CHCHD10* gene related diseases have been identified worldwide, they remain relatively rare. However, SMAJ is the most prevalent *CHCHD10*-linked disease globally, primarily due to its higher occurrence in the Finnish population (26). The existence of *CHCHD10*-linked diseases points to a direct link between mitochondrial function and motor neuron degeneration. Some mutations in *CHCHD10* have been shown to impair mitochondrial regulation, particularly in stressful situations, and to cause a less efficient ATP production due to increased proton leakage in mitochondria (4, 5, 8, 27). Nonetheless, the precise effects of *CHCHD10* variants on mitochondrial function are not fully understood (28), and only one previous study has explored the global metabolic consequences of CHCHD10 dysfunction in humans (10). In the present study, we conducted exercise testing on SMAJ individuals to gain insights into whether the metabolic response is like mitochondrial diseases caused by genetic defects directly targeting the respiratory chain, such as MELAS and CPEO. Through this investigation, we aimed to enhance our understanding of how *CHCHD10* variants affect mitochondrial metabolism *in vivo* in exercise associated stress situation.

In the SMAJ group, we observed a reduced maximal oxygen consumption, maximal power output, and reduced in oxygen pulse similar to the MELAS/CPEO group, indicating decreased oxidative capacity. Maximal oxygen consumption is considered a single comprehensive measure of aerobic or oxidative capacity (20, 22). A corresponding decrease in maximal oxygen consumption has been observed in different types of spinal muscular atrophy and has been shown to relate to the severity and prognosis of the disease (29–31). Furthermore, the ventilatory threshold in our study was lower in the MELAS/CPEO group and to a lesser extent also in the SMAJ group compared to the healthy controls corresponding to conditions limiting O_2_ delivery (32–34), and possible training effect in those healthy (34–36). As aerobic training is well-documented to improve maximal oxygen consumption (22), aerobic exercise could be useful in helping preserve the remaining oxidative capacity in these patients.

Lactate production increases during exercise as anaerobic energy metabolism increases. In mitochondrial myopathies, an elevated lactate concentration is typical both at rest and during exercise (16, 18, 19). On the other hand, in patients with ALS, lactate concentrations from capillary samples have been shown to be lower than in healthy controls in proportion to the reduction in the VO_2_ peak (37). In our study, the elevation in lactate typical of mitochondrial myopathies was observed in the MELAS/CPEO group, but in the SMAJ group the lactate levels were even lower than in healthy controls with a significant difference in repeated measurement tests. Similarly, in a recent study on biomarkers of SMAJ (10), no increase in lactate was observed in resting blood samples, but changes in pyruvate and succinate levels indicated that some metabolic changes related to mitochondrial dysfunction were present. These findings are in line with the scarcity of mitochondrial myopathy findings, such as COX-negative fibres, in muscle biopsies of individuals with SMAJ (38). As such, our results provide further confirmation that SMAJ does not exhibit features of mitochondrial myopathy, even under conditions of strenuous exercise. This discovery emphasizes the distinctions between disease-associated variants of *CHCHD10*. Notably, the p.Gly58Arg variant is associated with isolated mitochondrial myopathy, and some affected patients also exhibit elevated lactate levels (6, 39, 40) and mitochondrial myopathy has also been demonstrated in patients with the Ser59Leu variant (8).

An increase in lactate production during exercise also results in a rise in venous CO_2_, which can be measured as an increase in exhaled CO_2_ (V′CO_2_). In patients with severe mitochondrial disease, impairment of oxidative phosphorylation manifests with a decreased capacity of skeletal muscle to increase oxygen consumption (V′O_2_) because of increased dependence of on anaerobic metabolism leading to a relative increase in V′CO_2_ driven by increased lactate production (19). The increase in CO_2_ increases ventilation, in the present study seen as an increase in VE/V′O_2_ and in the significant decrease in FETCO_2_, findings observed in mitochondrial disease in previous studies (19, 41). These findings indicate an increase in ventilation in mitochondrial disease to be excessive and are common in hyperventilation (42, 43). In ALS, higher VE/V′O_2_ has also been shown to be an indicator of poor prognosis, even if it did not significantly differ from healthy controls (30), but these changes of FetCO_2_ and VE/V′O_2_ typically related to mitochondrial dysfunction in an exercise test were absent in the SMAJ group in our study.

The electrodiagnostic tests showed mild to severe motor neuropathy in all subjects with SMAJ and mild or moderate sensory neuropathy in some of them, as has been previously documented (1, 7). These results are in line with the established understanding of SMAJ, where the primary pathological feature involves the degeneration of spinal motor neurons, leading to secondary markers of muscle damage arising from neurogenic impairment (1, 10). CPEO and MELAS are known to cause myopathy, but both are known to also cause neuropathy in subsets of patients (44). In individuals with CPEO or MELAS studied here, we found no evidence of neuropathy, whereas findings of myopathy were prominent. As such, muscle weakness in our SMAJ patients is likely neurogenic in origin, whereas it is myogenic in individuals with CPEO or MELAS. Furthermore, different changes are likely in the cardiorespiratory regulation by the skeletal muscle reflexes in between these patient groups. In patients with sensory neuropathy, both the mechanoreflex and metaboreflex might be muted, whereas in metabolic myopathies the metabolic component of the muscle reflex has been suggested to be exaggerated due to increased lactate production and the concurrent drop in pH in the muscle, important mediators to the reflex (19, 45). The ratio of V′CO_2_ and V′O_2_ is the respiratory exchange rate (RER), a value that is commonly used as an indicator for the level of exercise maximality (46) but that can be exceptionally high in muscle mitochondrial disease (19, 41). In the patients with SMAJ, this kind of increase of ventilation was not found in the present patient material, which also suggests that a similar muscle metabolic defect as in PEO or MELAS does not exist in SMAJ.

### 4.1. Strengths and limitations

Given the rarity of the Finnish SMAJ mutation, a relatively small group of the disease was available and volunteered in the present study. To counter the small group size of the SMAJ disease, in addition to healthy controls, we also included controls suffering from a different form of muscle disease to better bring out the differences specific to SMAJ. Unfortunately, the sex distribution was uneven, with only four male subjects. In addition, there was some variation in the ages of the groups, but this was considered by adjusting the clinical profile of the 11 subjects. In addition, the patients were carefully diagnosed and examined, and despite the small number of patients, the findings are clear and characterize the exercise performance in SMAJ compared to healthy and diseased controls. Another limitation of the study was that the ammonia samples in the MELAS/CPEO group were performed with different analysing methods and had to be discarded from the analyses.

There was some variety in the maximality of the exercise between subjects, partly related to the need for blood sampling during cycling. However, the mitochondrial diseases are known to affect RER which is the relation of CO_2_ intake (V′CO_2_) to O_2_ consumption (V′O_2_) which increases in excessive lactate production such as in MELAS/PEO, so the assessment of maximality in these patients is not straightforward.

## 5. Conclusion

To our knowledge, this is the first report on cardiopulmonary exercise testing for subjects with SMAJ, or other predominately motor neuron form diseases caused by variants in *CHCHD10* gene. We observed a decrease in maximal oxidative capacity and maximal power output in SMAJ like in the mitochondrial disease group. However, the increased ventilation and rise in lactate known to be associated with mitochondrial myopathies were absent in SMAJ supporting the lack of mitochondrial myopathy in this disease.

## Data availability statement

The datasets presented in this article are not readily available because the Finnish law for research does not allow distribution of the data outside of the hospital.

## Ethics statement

The studies involving humans were approved by Medical ethic Board of Helsinki Uusimaa district. The studies were conducted in accordance with the local legislation and institutional requirements. The participants provided their written informed consent to participate in this study. Written informed consent was obtained from the individual(s) for the publication of any potentially identifiable images or data included in this article.

## Author contributions

NR: Formal analysis, Investigation, Visualization, Writing – original draft. EP: Investigation, Resources, Writing – review & editing. HL: Resources, Writing – review & editing. EY: Conceptualization, Writing – review & editing. RL: Formal analysis, Writing – review & editing. AS: Resources, Writing – review & editing. MA: Resources, Writing – review & editing, Conceptualization, Supervision. PP: Conceptualization, Formal analysis, Funding acquisition, Investigation, Resources, Supervision, Writing – original draft.
