# Supplementary material for: Lowered oxidative capacity in spinal muscular atrophy, Jokela type; comparison with mitochondrial muscle disease
Source: Front Neurol. 2023 Nov 8;14:1277944. doi: 10.3389/fneur.2023.1277944 (PMC10663357; doi:10.3389/fneur.2023.1277944)
Supplement: Supplementary file 1 [file Table_1.DOCX]

Supplemental Tables.

Table A 1. Plasma lactate and ammonia concentrations per diagnosis group

|  | **Lactate (mmol/l), Mean (SD)** | | | **Ammonia (µmol/mol), Mean (SD)** | |
| --- | --- | --- | --- | --- | --- |
|  | **SMAJ**  **n=11** | **MELAS/CPEO**  **n=26** | **Control**  **n=28** | **SMAJ**  **n=11** | **Control**  **n=28** |
| **Rest** | 1.01 (0.30) | 2.07 (1.21) | 0.94 (0.23) | 12.2 (9.4) | 10.6 (5.7) |
| **Light Exercise** | 1.25 (0.51) | 2.41 (1.49) | 1.09 (0.31) | 12.6 (8.0) | 14.0 (8.0) |
| **Maximal Exercise** | 4.75 (2.50) | 5.82 (2.63) | 5.52 (2.02) | 29.0 (15.3) | 32.8 (14.1) |
| **2 min into Recovery** | 7.20 (3.62) | 8.57 (2.55) | 8.62 (2.68) | 48.5 (27.9) | 49.8 (28.8) |
| **4 min** | 6.99 (3.30) | 9.25 (2.80) | 9.32 (2.70) | 48.3 (28.8) | 54.0 (26.1) |
| **6 min** | 7.76 (3.87) | 9.28 (3.12) | 9.31 (2.65) | 51.6 (33.8) | 49.6 (27.2) |
| **10 min** | 7.31 (3.55) | 9.00 (2.79) | 9.27 (2.58) | 42.9 (30.4) | 44.1 (28.1) |
| **20 min** | 6.07 (3.47) | 7.72 (2.53) | 6.96 (2.44) | 28.3 (21.8) | 28.0 (16.8) |
| **30 min** | 4.98 (3.08) | 6.23 (2.15) | 5.00 (1.78) | 19.9 (13.9) | 17.3 (11.4) |

SD= standard deviation. SMAJ = Spinal Muscular Atrophy, Jokela Type. MELAS/CPEO= mitochondrial disease group. (Ammonia values for MELAS/CPEO group were left out of analysis because the difference in analyser used).

Table A 2. Linear model results for plasma lactate and ammonia concentrations adjusted for age, sex, and BMI.

|  | **Lactate** | | | **Ammonia** | | |
| --- | --- | --- | --- | --- | --- | --- |
|  |  | **B(SE)** | **p-value** |  | **B(SE)** | **p-value** |
| **Rest** | Control | 0 | reference | Control | 0 | reference |
|  | SMAJ | 0.072 (0.35) | 0.83 | SMAJ | 1.14 (2.36) | 0.63 |
|  | **MELAS/CPEO** | **1.30 (0.27)** | **<0.001** |  |  |  |
| **Light Exercise** | Control | 0 | reference | Control | 0 | reference |
|  | SMAJ | 0.16 (0.34) | 0.63 | SMAJ | -1.43 (2.78) | 0.61 |
|  | **MELAS/CPEO** | **1.33 (0.27)** | **<0.001** |  |  |  |
| **Maximal Exercise** | Control | 0 | reference | Control | 0 | reference |
|  | SMAJ | -0.52 (0.77) | 0.50 | SMAJ | -1.98 (4.30) | 0.65 |
|  | MELAS/CPEO | 0.092 (0.60) | 0.88 |  |  |  |
| **2 min into Recovery** | Control | 0 | reference | Control | 0 | reference |
|  | SMAJ | -1.09 (0.84) | 0.20 | SMAJ | 1.55 (8.63) | 0.86 |
|  | MELAS/CPEO | -0.61 (0.68) | 0.37 |  |  |  |
| **4 min** | Control | 0 | reference | Control | 0 | reference |
|  | SMAJ | -1.66 (0.87) | 0.058 | SMAJ | -2.42 (8.00) | 0.76 |
|  | MELAS/CPEO | -0.49 (0.66) | 0.45 |  |  |  |
| **6 min** | Control | 0 | reference | Control | 0 | reference |
|  | SMAJ | -1.22 (0.92) | 0.19 | SMAJ | 3.87 (9.16) | 0.67 |
|  | MELAS/CPEO | -0.26 (0.71) | 0.71 |  |  |  |
| **10 min** | Control | 0 | reference | Control | 0 | reference |
|  | SMAJ | -1.73 (0.89) | 0.051 | SMAJ | -0.80 (9.63) | 0.93 |
|  | MELAS/CPEO | -0.48 (0.69) | 0.49 |  |  |  |
| **20 min** | Control | 0 | reference | Control | 0 | reference |
|  | SMAJ | -0.78 (0.86) | 0.37 | SMAJ | -0.13 (5.5) | 0.98 |
|  | MELAS/CPEO | 0.57 (0.68) | 0.40 |  |  |  |
| **30 min** | Control | 0 | reference | Control | 0 | reference |
|  | SMAJ | -0.0004(0.71) | 1.00 | SMAJ | 2.5 (3.83) | 0.46 |
|  | **MELAS/CPEO** | **1.17 (0.57)** | **0.041** |  |  |  |

B= Estimated regression coefficient for one-unit increase in continuous variables. SE=Standard error. SMAJ = Spinal Muscular Atrophy, Jokela Type. MELAS/PEO= mitochondrial disease group. (Ammonia values for MELAS/PEO group were left out of analysis because the difference in analyser used). Statistically significant results (p<0.05)are in bold. BMI=body mass index.
